# A phenomenon in urban disruption: the emergence of Coworking Spaces in Bandung

**DOI:** 10.1016/j.heliyon.2021.e07663

**Published:** 2021-07-24

**Authors:** Ridwan Sutriadi, Dimas Muhammad Fachryza

**Affiliations:** Urban and Regional Planning, School of Architecture, Planning, & Policy Development (SAPPD), Institut Teknologi Bandung, Jl. Ganesha No. 10 Kota Bandung 40132, Provinsi Jawa Barat, Indonesia

**Keywords:** Coworking spaces, Urban disruption, Spatial patterns, Agglomeration economies, Bandung city

## Abstract

Since the emergence of urban planning science at the beginning of 20^th^ century, urban disruption as innovation is highly connected to the urban planning framework, it goes along in responding urban problem, in conjunction with the civilization development, from innovation in terms of planning process and field survey in the early 20^th^ century to the use of information and communication technology (ICT) as analytical planning tools in 2010s. The emergence of Coworking Spaces in Bandung, a capital city of West Java where many universities were founded, and a place for youth scholarship and creativity, is an appropriate location to be used as a case study. This paper aims to analyze spatial patterns and linkages with local features, using kernel density and also somer's d association analysis to determine the presence, strength, and direction of the relationship asymmetrically. This is important to analyze the emergence of Coworking Spaces as a part of urban disruption process in promoting city centre vitality while the advance of technology, service sector expansion, liveable building design become primary focus of globally Coworking Spaces discourses.

## 1 Introduction

The advent of the 21^st^ century resulted in rapid information, communication, and technology (ICT) growth. Modern lifestyles and economic systems have been affected by this, including new job models and urban planning aspects (Delitheou et al., 2019; Hendarman and Tjakraatmadja, 2012; Hsu et al., 2008; Huliana and Ellisa, 2019; Moriset, 2013; Schürmann, 2013; Yigitcanlar, 2010). The world has undergone major economic transformation, especially in the 18^th^ century, from the agricultural economy to the industrial economy. Followed by the urban spatial structure transformation since 1960s (Friedmann and Miller, 1965; Kloosterman and Musterd, 2001; Sutriadi and Miftah, 2020). Then, the concept of an economic transformation to a knowledge-based economy (KBE) originated in the late 20^th^ century (Carlaw et al., 2006; Hendarman and Tjakraatmadja, 2012; Jamal, 2018; Švarc and Dabić, 2017).

ICT also affects social aspects, especially for digital natives, a community that lives with technology and has characteristics that are brave against conventional cultures (Hsu et al., 2008; Huliana and Ellisa, 2019). Today, digital natives will become digital nomads who have the chance to work anywhere and at any time, including from home (Reichenberger, 2018; Thompson, 2019). However, to avoid the issue of social isolation and limitations in working with others, digital nomads need 3^rd^ place for socialization.

As a consequence of demands for modern model workstyles, the rise of Coworking Spaces (Huliana and Ellisa, 2019; Schürmann, 2013). The concepts went through the hybridization process of the 3^rd^ place workspaces (Fost, 2008; Moriset, 2013). In order to socialize informally but remain productive, Coworking Spaces build a new hope to break through the issue of social isolation (Moriset, 2013; Schürmann, 2013; Waters-Lynch et al., 2016). Coworking Spaces can also be seen as a modern social infrastructure because it gives rise to different interaction patterns with home (1^st^ places) and conventional offices (2^nd^ places) as well as knowledge-based economic accelerators in order to improve competitiveness and comparative advantages (Jamal, 2018; Merkel, 2015; Moriset, 2013).

There is a good response to the concept of Coworking Spaces, particularly from community and start-ups, as well as large-scale businesses (Anggraeni, 2019; Brown, 2017; Lang LaSalle, 2016; Parrino, 2015; Schürmann, 2013). Therefore, Coworking Spaces are growing exponentially and becoming a global phenomenon and spread worldwide, including the pioneer Coworking Spaces in Indonesia was established in Bandung (Kresna, 2016; Mariotti et al., 2017). These phenomena and a new journey of Coworking Spaces in Bandung, Indonesia can be used to prepare the new economic era, KBE (Carlaw et al., 2006; Hsu et al., 2008; Jamal, 2018; Merkel, 2015; Moriset, 2013; Švarc and Dabić, 2017).

The term Coworking Spaces in spatial planning documents is still unfamiliar and unpracticed. However, the massive development and spatial patterns of Coworking Spaces can affect the internal urban structure (Huliana and Ellisa, 2019; Mariotti et al., 2017; Moriset, 2013). This paper aims to analyze spatial patterns and linkages with local features to understand the basic phenomena of the development of Coworking Spaces in Bandung which can be an additional feature of consideration in the formulation of spatial planning documents. The scope of this research is Bandung, a capital city of West Java, Indonesia where many universities were founded, and a place for youth scholarship and creativity is an appropriate location to be used as a case study.

In the past, the city of Bandung was the epicenter of Indonesian telecommunications technology growth (Ranawati, 2020; The Malabar Project, 2012). In addition, Bandung is a member of the UNESCO Creative Cities Network and offers an interconnection to the rest of the world through direct flights (UNESCO, 2015). Thus, new technologies can quickly reach the market and spread rapidly which is backed by a group of young people who enjoy trying new things, including Urban Distruption in the form of Coworking Spaces. These provides a unique reason for research in this area.

**Figure 1:**
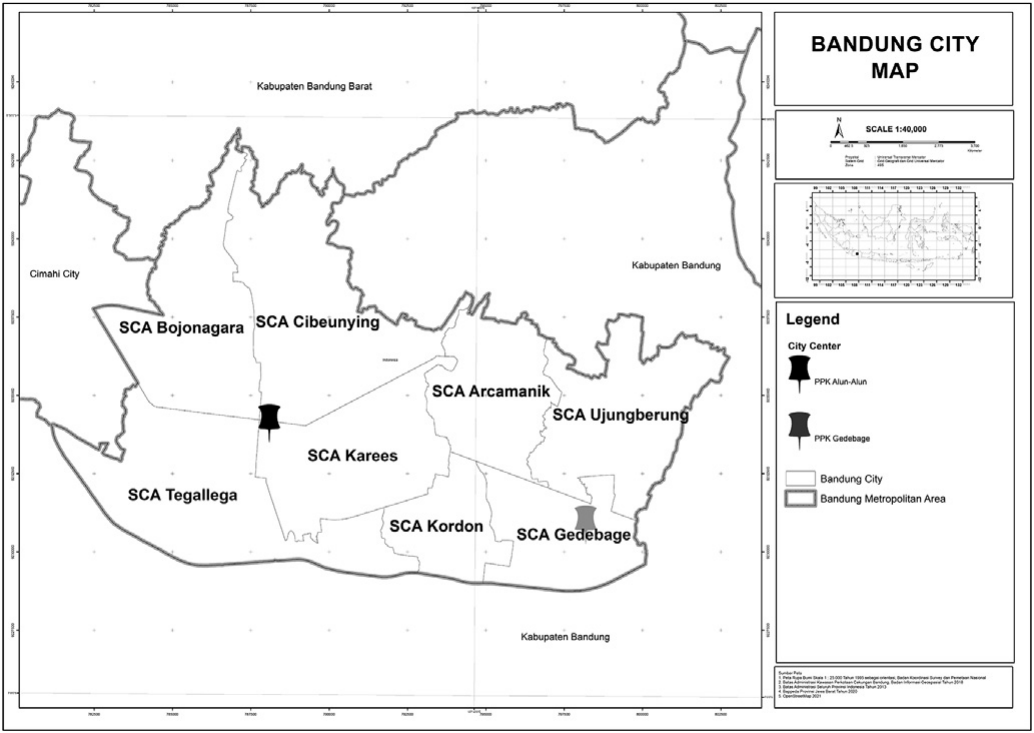
Bandung city map. Source: Geospatial Information Bureau.(2020)

Based on Bandung City Spatial Plan - RDTR 2015-2035, the internal urban structure of Bandung City is divided into two areas based on the City Center, namely PPK Alun-Alun in the West Bandung Area (WBA) and PPK Gedebage in the East Bandung Area (EBA). WBA consists of four Sub-City Area (SCA), namely SCA Bojonagara, SCA Cibeunying, SCA Tegallega, and SCA Karees. Furthermore, EBA consists of four SCA, namely SCA Arcamanik, SCA Ujungberung, SCA Kordon, and SCA Gedebage (see Figure 1).

This paper begins by providing a general overview of the context, study definitions, and methodology. The literature review, discussion of the knowledge-based economy, a brief of Coworking Spaces, agglomeration economies, disruption, and urban policy are all covered in the following section. The third part is an is analysis to answer the objectives. The article concludes with conclusions and suggestions based on the study's findings, which can be used as guidance for future studies as well as lessons learned for other cities.

## 2 Literature review

This literature review highlights several topics, from the emergence of the knowledge-based economy (KBE) to previous research related to Coworking Spaces in the context of urban planning and development, by considering the emergence of Coworking Spaces as a new phenomenon in Bandung city.

### 2.1 Knowledge-based economy (KBE)

The Organization for Economic Co-Operation (OECD) initiated the concept of global economic restructuring towards a knowledge-based economy (KBE) in 1996 after deindustrialization in the 1960s to 1980s (Carlaw et al., 2006; Hendarman and Tjakraatmadja, 2012; Švarc and Dabić, 2017). The idea was initiated in the Lisbon Strategy (Lisbon I) in 2000, followed by a relaunched strategy (Lisbon II) in 2010 (Švarc and Dabić, 2017). The KBE era has emerged as a key trend in global economic growth in the twenty-first century, and cities must adapt good strategies and policies to win dynamic global competition by enhancing the quality of life in order to attract knowledge-based workers and knowledge societies (Hsu et al., 2008; Yigitcanlar, 2010).

KBE is the process of creating, distributing, and utilizing information in order to create knowledge communities and increase access to knowledge (Carlaw et al., 2006; Yigitcanlar, 2010). The main assets are shifted from labor, machine, and land to the creativity and innovations (Hendarman and Tjakraatmadja, 2012; Hsu et al., 2008; Švarc and Dabić, 2017). This has been an impetus for developing countries to compete globally by creating commercialized innovations in the coming decades by configuring land use (ADB, 2014; Carlaw et al., 2006; Hsu et al., 2008; Seto and Pandey, 2019; Stevens and Khan, 2018; Švarc and Dabić, 2017; Yigitcanlar, 2010).

### 2.2 Agglomeration economies

The agglomeration economies were first proposed as one of the economic geographic methods in the 1980s by Alfred Marshall. In this schema, the phenomenon of firms located in the same region would obtain economies of scale, and even economic specialization can be achieved. If the firms in the cluster do not have economies of scale, they tend to be dispersed or seek other equilibrium locations (McCann, 2013).

Marshall provides three reasons why such economies of scale must be achieved. The first reason is about knowledge spillovers which is the proximity as influence factors for effective and efficient knowledge transfer, especially in tacit information or incomplete knowledge. The second reason is about non-traded local inputs which is the proximity as factor to open the possibilities for certain specialist inputs to be provided in the cluster. The final reason is about local skilled labor pool which is the proximity will make firms easier to expand and share its labor force (McCann, 2013). There are three types or classification in order to describe the particular nature of agglomeration economies, namely internal returns to scale (occur on internal scale), localization economies (occur on the same sector cluster scale), and urbanization economies (occur on city scale) (McCann, 2013).

Following the study of the agglomeration of firms, there is emerging study of people clustering through the creativity class hypothesis by Florida (2012) and urban consumption by Glaeser (2001). The hypothesis of the creativity class explains that creative individuals appear to be found in the most desirable places. The urban migration of creative people will create an agglomeration of professional people and foster urban creativity (Florida, 2012; McCann, 2013). These creative hotspots have become the key drivers of urban policies to promote the growth of knowledge-based (Mengi et al., 2020). A related point regarding urban consumption is that professional people with high incomes have the capacity to move with quality atmosphere to the great city, such as restaurants, coffee shops, museums, theaters, etc. This hyphotesis views modern cities and large cities with intact facilities as a place of work as well as recreation (McCann, 2013).

### 2.3 Brief of Coworking Spaces

The context for the emergence of the idea of Coworking Spaces is the need for new workstyles as a result of various technological advances (Huliana and Ellisa, 2019; Moriset, 2013; Schürmann, 2013). Coworking Spaces are a new urban phenomenon that reflects a shift in knowledge workers toward flexible, project-based tasks and the freedom to work independently in a different location as a result of deeper social changes (Nakano et al., 2020; Richardson, 2017). Coworking Spaces are a type of collaborative workspace where people can work independently or as part of a team to share uncommon equipment, ideas, and information (Jamal, 2018; Schürmann, 2013). Kwiatkowski and Buczynski (2011) can also describe Coworking Spaces based on 5 principles, namely collaboration-community-sustainability-openness-accessibility (Schürmann, 2013; Kwiatkowski and Buczynski, 2011).

In 1999, Bernard de Koven introduced the term “coworking” to describe new working style through “working-alone-together” to facilitate an equal collaborative work atmosphere (Orel and Dvouletý, 2020; Parrino, 2015). The term "coworking" refers to the cooperation and sharing of work space between individuals who work independently given the reciprocal relationships formed on the basis of spontaneous or moderate processes in temporary or permanent collaborative work spaces (Orel and Dvouletý, 2020). Then, Neuberg was the first individual to associate the word “coworking” with flexible workspaces and collaborative use through the establishment of the small workspace at Spiral Muse, San Francisco in 2005 to treat problems of social isolation (Capdevila, 2015; Di Risio, 2019; Garrett et al., 2017; Orel and Dvouletý, 2020; Parrino, 2015; Schürmann, 2013; Spinuzzi, 2012). After almost a year, Neuberg relocated and renamed the workspace to “Hat Factory” which is known as the world's first full-time coworking space (Di Risio, 2019; Parrino, 2015; Schürmann, 2013).

The first coworking space is taking steps to achieve active momentum and wider recognition, an individual movement that popularizes coworking as a model for the use of flexible workspaces (Orel and Dvouletý, 2020). In 2005 and 2010 were the periods of origin and development of the coworking spaces model followed by the popularization period of 2010–2014 (Fost, 2008; Moriset, 2013; Orel and Dvouletý, 2020). Further hybridisation of adopting the terms incubator for startups, business centers and telecentres from the model began in 2014 as an ongoing process (Moriset, 2013; Orel and Dvouletý, 2020).

The application of Coworking Spaces can adjust the market needs and preferences of coworking as long as in accordance with the 5 principles. The reasons why Coworking Spaces are chosen is 1) affordable rental cost and promos; 2) a social & enjoyable atmosphere; 3) an interaction & collaboration; 4) a clean workspaces & good services; 5) 24/7 access or flexible worktimes; 6) workshops & events; 7) friend or company contract; 8) basic and special office infrastructure; and 9) strategic location (Cabral & van Winden, n.d.; Dianovita and Khoirunurrofik, 2020; Mariotti et al., 2017; Rutten, 2003; Weijs-Perrée et al., 2019; Zhou, 2019).

### 2.4 Coworking Spaces, disruption, and urban policy

Several literatures have seen the emergence of new forms of office space in accordance with the latest working styles, which are seen as ideal places to work as well as a source of social support for individual practitioners and collective group innovations as physical entitie (Brown, 2017; Moriset, 2013; Orel and Dvouletý, 2020; Reichenberger, 2018; Schürmann, 2013; Thompson, 2019). Coworking spaces growth is a symptom of disruption to technology, markets (responsive to demand), and established regulatory mechanisms that is caused by technical developments or structural changes in periods of turmoil (Babb et al., 2018; Davies et al., 2017; Martin, 2016). Apart from disrupting the market, planners and regulators are often confused about adapting to the dynamics that occur (Babb et al., 2018; Nakano et al., 2020). Therefore, most municipal policies and land use legislation have not adapted to the Coworking Spaces phenomenon that can bring legal confusion and impede initiatives (Babb et al., 2018; Nakano et al., 2020).

Coworking Spaces can support job creation, wealth and increase technology transfer and innovation so that it can be recommended and integrated in public policy (Babb et al., 2018; Buksh and Mouat, 2015; Hicks and Faulk, 2018; Schmidt et al., 2014; Schmidt and Brinks, 2017). However, their contribution has not been verified because the current empirical evidence is not strong yet, due to the rare regulations and unclear implementation (Avdikos and Merkel, 2020; Babb et al., 2018; Nakano et al., 2020). Urban planners and regulators have usually interfered by means of zoning instruments. However, this instrument is aimed at assessing categories of land use and licensing problems, so they often neglect work intensity, such as worker density per space (Babb et al., 2018).

Traditionally, Planners rely on land use policies, zoning regulations and development assessments to support efficient functioning. However, the nature of urban land and labor markets has changed due to this disruption. This implies that planners who rely solely on limited measures to organize workspaces need to reflect on the effectiveness of current instruments and practices, otherwise there will be a risk of policies becoming obsolete (Babb et al., 2018). In simple terms, planners and regulators must be adaptive and ready to face the dynamics of the emerging nature of urban spaces. Finally, in order to achieve optimal urban objectives, the planning process must change rather than merely reduce negative externalities in land use markets aimed at securing potential gains for private and public interests (Babb et al., 2018; Gore et al., 1989; Rydin, 2011). In this dynamic situations, planners can involve technology and increasing community participation in urban planning, such as collecting data, making maps, proposing ideas and, eventually approving or rejecting design proposals (Delitheou et al., 2019).

### 2.5 Previous research

There is Tobler's first law of geography: “everything is related to everything else, but near things are more related than distant things (Tobler, 1970). This law can explain the basic understanding of Coworking Spaces's emergence and spatial patterns phenomena.

There have been several previous studies discussed about spatial patterns and distribution of Coworking Spaces, one of which is research by Mariotti et al. (2017), entitled “Co-working Spaces in Milan: Location Patterns and Urban Effects”. Based on these empirical studies, the spatial patterns of Coworking Spaces in Milan associated with the proximity to potential market or business activity, college/universities or research centers, and intact infrastructure (public transportation, etc.) (Mariotti et al., 2017). Furthermore, Zhou (2019), entitled “Coworking as an emerging urban lifestyle: location analysis of coworking spaces in Manhattan, NYC”. The result of this exploratory research suggest that the current locations of Coworking Spaces in NYC are clustered and statistically significant correlations with coffee shops, groceries, drinking spaces, fitness center, museums, and subway stations to take more benefit from easy access to urban resources and its facilities (Zhou, 2019).

## 3 Methodology

This paper explores the association between Coworking Spaces and local features as an attempt to explore Coworking Spaces in the planning document. Therefore, this research uses a quantitative approach, kernel density and also using Somer's D association analysis.

Kernel density is an ArcGIS Pro analysis tool that calculates the density of features (points or lines) across raster cells. The kernel function is based on the Silverman mathematical function, which allows each adjacent curve function to be added together to form a density modeling function (ESRI, 2020; Wicklin, 2016). This research tool is required to understand the internal urban structure, including Coworking Spaces and local features. Additionally, Somer's D Association analysis is a form of bivariate analysis that uses discrete ordinal data types in associations (Healey, 2012; Wagner, 2015). This method is used to assess the presence, strength, and direction of an asymmetrical relationship (dependent and independent variables). The statistical result of +1 value shows that there is a deterministic and unidirectional relationship which means that all variations in the independent variable are taken into account in the dependent variable, vice versa (Wagner, 2015).

The density of Coworking Spaces is treated as a dependent variable in the analysis, while local features are treated as independent variables. The local features used in this study refer to Tobler's first law of geography, which states that "everything is related to everything else, but near things are more related than distant things" (Tobler, 1970). It also follows Florida's (2012) creativity class hypothesis and Glaeser's (2001) urban consumption hypothesis, in which local features such as restaurants, coffee shops, museums, theaters, etc. which triggered the creation of creative hotspots (Glaeser's (2001); Florida, 2012; McCann, 2013).

Furthermore, from the viewpoint of urban planners in Indonesia, land use policies and zoning laws must be considered, including the spatial pattern or distribution of spatial usage in an area which includes spatial designation for natural protection functions and spatial designation for business & social activities functions (Law No. 26 of 2007 on Spatial Planning, 2007). It is also an argument for choosing local features to be used, based on previous studies by Marirotti et al. (2017) and Zhou (2019). Thus, the local features in this paper are divided into five aspects:

1. Infrastructures: transportations, hospitals, college/universities, government officies, and sports & parks facilities;
2. Lifestyles: shopping centers, coffee shops, food services, and bar & pubs;
3. Tourism: hotels, discovery & events, and bank;
4. Socio-Geography: total population, population density, age (15–64), and education level (above high school);
5. Land use & Spatial Plan Areas.

**Figure 2:**
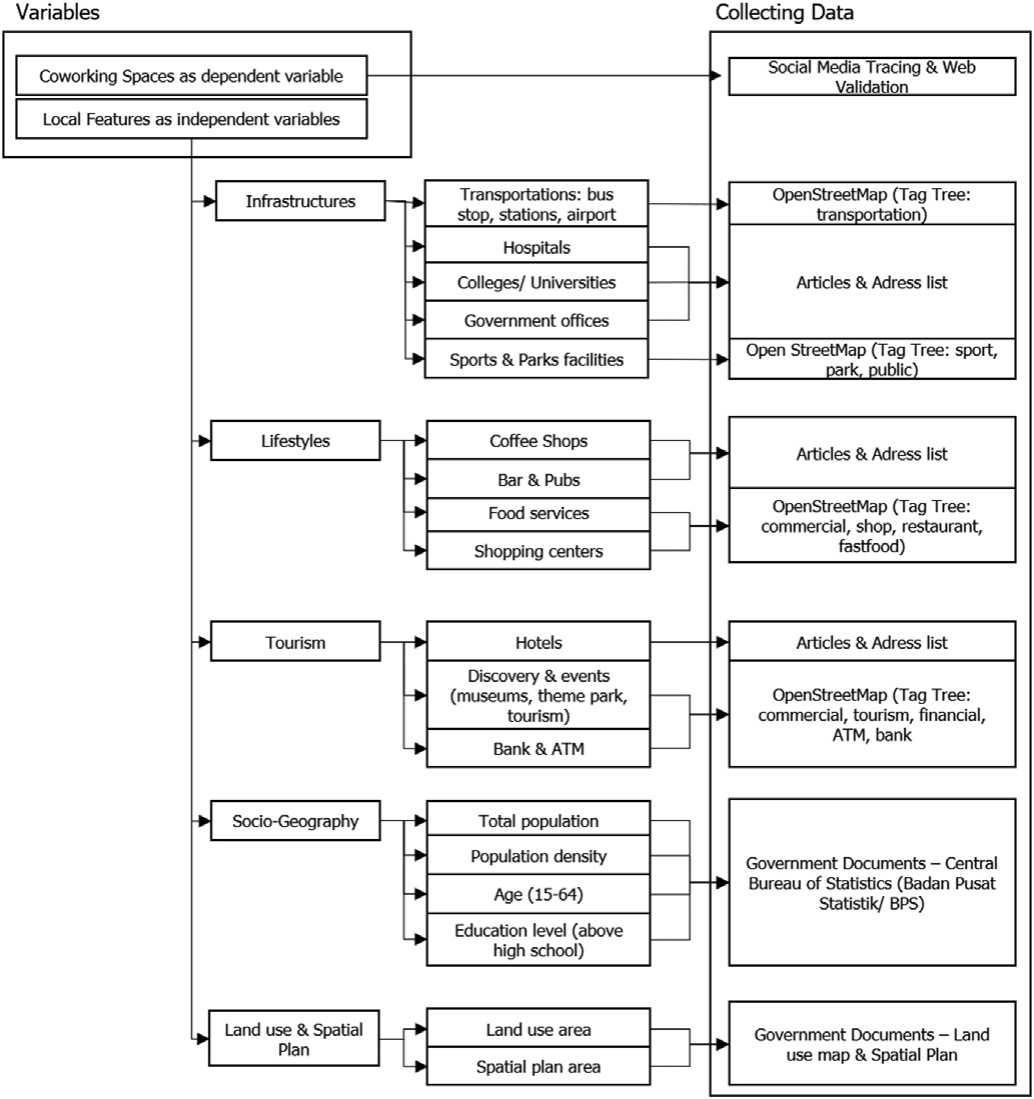
Method & collecting data. Source: Author, 2020.

This paper using secondary collection data from OpenStreetMap (OSM), google maps, social media, and related articles (see Figure 2).

## 4 Analysis

A discussion comprises into urban internal structure as well as a relation between coworking spaces density and local features.

### 4.1 Urban internal structure: Coworking Spaces and local features

1. Coworking Spaces Density

**Figure 3:**
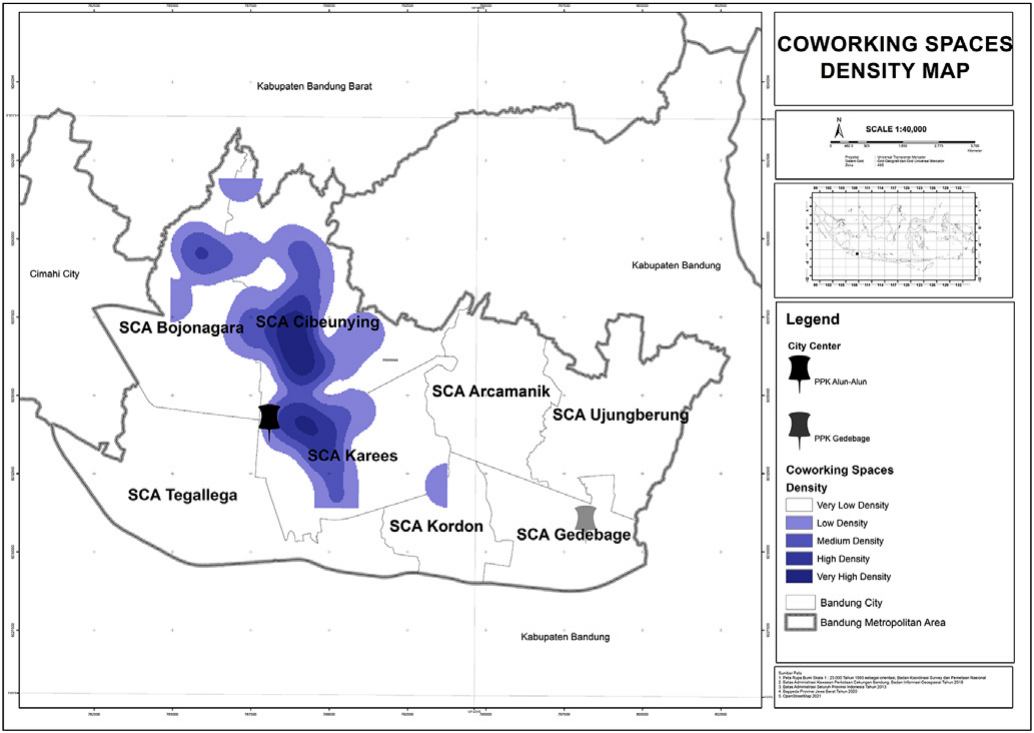
Coworking Spaces density. Source: Analysis, 2020.

Based on Figure 3. Coworking Spaces density, The spatial patterns of coworking spaces tend to form a clusters in West Bandung Area (WBA), especially SCA Cibenying and SCA Karees.

2. Local Features: Infrastructures, Lifestyles, Tourism, and Socio-Geography

Based on Table 1. Local Features, in general all of local infrastructure features are relatively centralized in West Bandung Area (WBA). These local features are: 1) Transportation infrastructures density: the concentration of transportation infrastructures (including the presence of bus stops, stations, and airport) is relatively centralized in WBA, especially in SCA Bojonagara, SCA Cibeunying, and SCA Karees; 2) Hospitals density: the concentration of hospitals is relatively centralized in WBA, especially in SCA Bojonagara and SCA Cibeunying; 3) College/university density: the concentration of college/university is relatively centralized in WBA, especially in SCA Bojonagara and SCA Cibeunying; 4) Government offices density: the concentration of government offices is relatively centralized in WBA, especially in SCA Cibeunying; and 5) Sports & Parks facilities density: the concentration of Sports & Parks is relatively centralized in WBA, especially in SCA Cibeunying.

**Table 1:** Urban local features.

Variables		West Bandung Area (WBA)				East Bandung Area (EBA)			
		SCA Bojonagara	SCA Cibeunying	SCA Tegallega	SCA Karees	SCA Arcamanik	SCA Ujungberung	SCA Kordon	SCA Gedebage
Infrastructures	transportations	v	v		v				
	hospitals	v	v						
	college/universities	v	v						
	government officies		v						
	sports & parks		v						
Lifestyles	shopping center	v	v	v	v				
	coffee shops		v						
	food services	v	v						
	bar & pubs		v						
Tourism	hotels	v	v		v				
	discovery & events	v	v	v	v				
	bank	v	v					v	
Socio-Geography	total population	v	v	v	v			v	
	population density	v		v	v				
	age (15–64)	v	v	v	v		v	v	
	education level (above high school)	v							

The distribution of all lifestyles facilities are also tends to WBA. These local features are 1) Shopping center density: the concentration of shopping center is relatively centralized in WBA (SCA Bojonagara, SCA Cibeunying, SCA Tegallega, and SCA Karees); 2) Coffee shops density: the concentration of coffee shops is relatively centralized in WBA, especially in SCA Cibeunying; 3) Food services density: the concentration of food services (including restaurant and fast food) is relatively centralized in WBA, especially in SCA Bojonagara and SCA Cibeunying; and 4) Bar & pubs density: the concentration of bar & pub is relatively centralized in WBA, especially in SCA Bojonagara and SCA Cibeunying.

Then, local features of tourism and socio-geography also exhibit the same phenomenon. 1) Hotels density: the concentration of hotel is relatively centralized in WBA, especially in SCA Bojonagara, SCA Cibeunying, and SCA Karees; 2) Discovery and events density: the concentration of discovery and events is relatively centralized in WBA (SCA Bojonagara, SCA Cibeunying, SCA Tegallega, and SCA Karees); 3) Bank density: the concentration of discovery and events is relatively centralized in WBA, especially in SCA Bojonagara and SCA Cibeunying. Furthermore, there is concentration in East Bandung Area (EBA), precisely in SCA Kordon; 4) Total population: the concentration of population is relatively located in WBA, especially in SCA Bojonagara and SCA Cibeunying. Furthermore, there is concentration in EBA, precisely in SCA Kordon; 5) Population density: the concentration of population density is relatively centralized in WBA, especially SCA Bojonaga, SCA Tegallega, and SCA Karees; 6) Age (15–64): the concentration of population – age (15–64) as a representative of workforce is relatively located in WBA (SCA Bojonagara, SCA Cibeunying, SCA Tegallega, and SCA Karees). Furthermore, there is concentration in EBA, especially in SCA Ujungberung and SCA Kordon; and 7) education level (above high school): the concentration of population – education level (above high school) as a representative of knowledge-society is relatively located in WBA, especially SCA Bojonagara.

3. Local Features: Land Use and Spatial Plan

**Figure 4:**
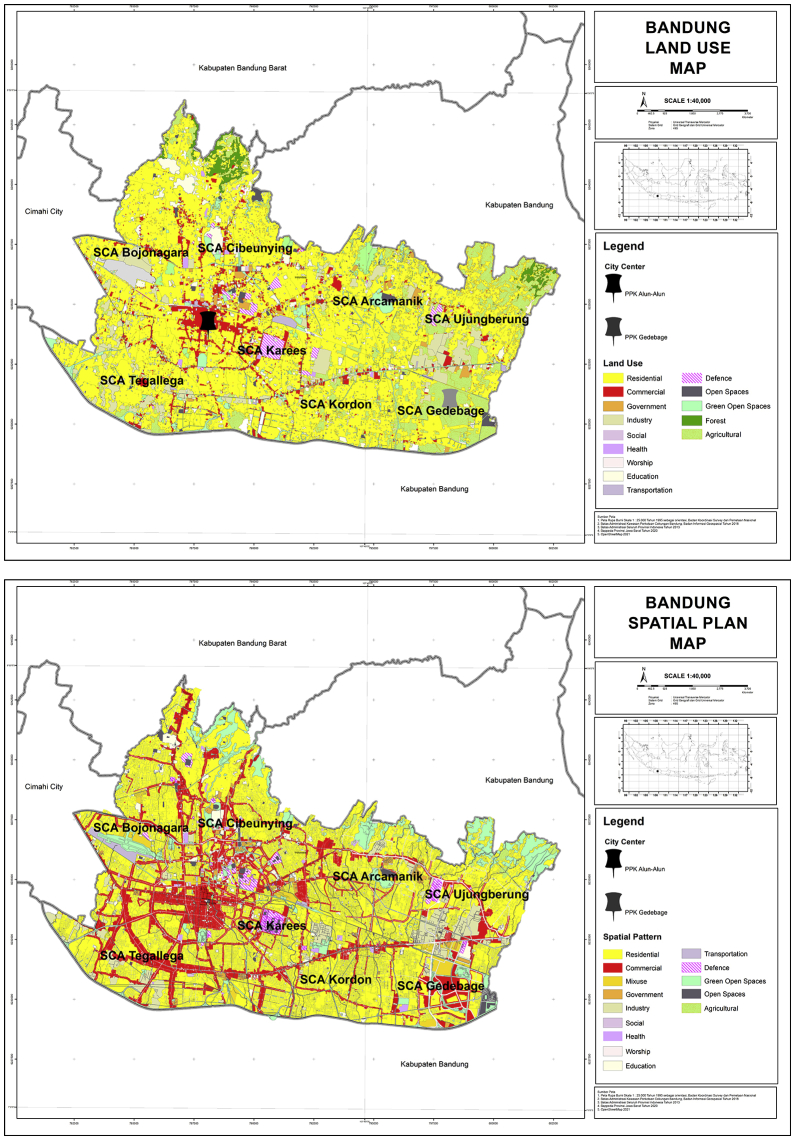
Land use and spatial plan. Source: Bandung Planning Agency (2016).

Based on Figure 4. Land Use and Spatial Plan, The majority land use in Bandung is residential areas (±56 %), agriculture (±11 %), and industry (±7 %). In internal urban structure perspective, land use area for airport, forest, industry, government, residential, education, defence, transportation, commercial, open spaces, health, and social areas tend to be in West Bandung Area (WBA).

While unproductive land use such as agriculture, cemetery, and worship area tend to be in East Bandung Area (EBA). Then, the majority spatial plan in Bandung is planned to have a high density residential (±25 %), medium density residential (±23 %), linear commercial area (±15 %), and low density residential (±10 %). In internal urban structure perspective, spatial plan area for defence, medium intensity for mix land use, traditional commercial area, shopping center, linear commercial area, government, private green spaces, education, health, worship places, transportation area tend to be in West Bandung Area (WBA). While spatial plan for agriculture, high intensity mix land use, industry, medium & low density residential, social culture tend to be in East Bandung Area (EBA).

### 4.2 Analysis association of the Coworking Spaces density and local features

Refers to previous analysis step, it can be classified and converted into ordinal scales (very low density = 1; low density = 2; medium density = 3; high density = 4; very high density = 5). Then, it can be used as input into somer's d association analysis between Coworking Spaces density and local features. Then, based on 66 local features are treated as independent variables, there are four local features that are statistically significant and has strong relationship.

1. Coffee Shops

Somer's d of coffee shops is statistically significant and has strong value (0.73) with coworking spaces density treated as the dependent variable. It can be interpret that 73 % of the variation in coworking spaces density can be accounted for by the variation in café & coffee shops. Errors of prediction are reduced by one-quarter (see Table 2).

2. Bar & pubs

**Table 2:** Association analysis result.

No	Variable	Value	
1	Coffee Shops	0.73	Strong-Positive
2	Bar & Pubs	0.719	Strong-Positive
3	Spatial Plan – Education Area	0.634	Strong-Positive
4	Sport & Parks Facilities	0.603	Strong-Positive

Somer's d of bar & pubs is statistically significant and has strong value (0.719) with coworking spaces treated as the dependent variable. It can be interpret that 71.9 % of the variation in coworking spaces density can be accounted for by the variation in bar & pub. Errors of prediction are reduced by almost one-quarter (see Table 2).

3. Spatial Plan – Education Area

Somer's d of spatial plan – education area is statistically significant and has strong value (0.634) with coworking spaces treated as the dependent variable. It can be interpret that 63.4 % of the variation in coworking spaces density can be accounted for by the variation in spatial plan – education area. Errors of prediction are reduced by one-third (see Table 2).

4. Sport & Park Facilities

Somer's d of sport & park facilities is statistically significant and has strong value (0.603) with coworking spaces treated as the dependent variable. It can be interpret that 60.3 % of the variation in coworking spaces density can be accounted for by the variation in sport & park facilities. Errors of prediction are reduced by one-third (see Table 2).

## 5 Conclusion

The phenomenon of Coworking Spaces as a form of urban innovation has arrived in Bandung City and clustered in West Bandung Area (WBA), especially in SCA Cibeunying and SCA Karees. It shows a new opportunity for modern forms of work during the digital transformation period in urban areas with higher and diverse population densities compared to non-urban areas. This is also an opportunity to achieve economies of scale advantage in knowledge spillovers, sharing local inputs, and local skilled labor pool at local scale. Although the rationale and concept are still unclear how much the contribution to the city's economic growth will be, because it begins from following the global cities trend.

There are several local features that statistically significant and has strong affect to the spatial pattern of Coworking Spaces such as proximity to coffee shops, bar & pubs, spatial plan – higher education area (related to universities or research centers), and sport & park facilities. This phenomenon is in accordance with the people's clustering – creative class and consumer city hypothesis which describes how innovative people would be attracted and move to a places with appropriate infrastructure and lifestyle facilities. Furthermore, related to previous studies of Coworking Spaces abroad, indeed the presence of Coworking Spaces cannot be separated from the existence of universities as scientific centers. As we all know, Bandung is famous for its many public and private universities, but it is expected that more studies have to be developed to show that the presence of Coworking Spaces in this city is triggered or correlated by the existence of these universities, especially related to research and development activities developed by existing universities and collaboration among universities with the market as well as with global networks.

However, in terms of Bandung, adjustment is needed in responding to local problems, especially to make Bandung more competitive. Since Coworking Spaces involves collaboration, it is also necessary to develop a wider network of collaboration between stakeholders for the common interest in promoting a strong, inclusive, and sustainable city. Then, Coworking Spaces in Bandung is seen not just as a disturbance in main activity accessories, but also as a disruption in increasing citizen's knowledge and skills that foster mutual value towards a new economic era, Knowledge-based economy and compete globally.

## 6 Recommendation

The phenomenon of Coworking Spaces emerged after Bandung had a spatial plan document, both general and city plans, so it is necessary to justify the role of Coworking Spaces in the transformation of the structure and spatial pattern of the City of Bandung. There needs to be a specification for the description of Coworking Spaces in terms of spatial planning for a more detailed level than the detailed spatial plan, namely at the level of urban design guidelines (RTBL – Rencana Tata Bangunan dan Lingkungan). This will make it possible to formulate a Coworking Spaces not only in relation to its activities, but also with a design that is in accordance with the theme or objective of its urban design guidelines to maintain consistency at the whole level, from macro to micro spatial policies, in planning documents.

In relation to the recommendation for stakeholders and policy makers is how to respond to the presence of this Coworking Spaces phenomenon, because even though it occurs at the city level, its impact and ICT infrastructure network are related to the national policy level. Furthermore, how is the position in deciding Coworking Spaces at the city level in the realm of the new planning permission policy in Indonesia as a continuation of a new law on Omnibus Law, which is called the Confirmation of Spatial Utilization Activities (KKPR – Kesesuaian Kegiatan Pemanfaatan Ruang).

## Declarations

### Author contribution statement

Ridwan Sutriadi: Conceived and designed the experiments; Analyzed and interpreted the data; Contributed reagents, materials, analysis tools or data; Wrote the paper.

Dimas Muhammad Fachryza: Conceived and designed the experiments; Performed the experiments; Analyzed and interpreted the data; Contributed reagents, materials, analysis tools or data; Wrote the paper.

### Funding statement

This research did not receive any specific grant from funding agencies in the public, commercial, or not-for-profit sectors.

### Data availability statement

Data will be made available on request.

### Declaration of interests statement

The authors declare no conflict of interest.

### Additional information

No additional information is available for this paper.

## References

[bib1] ADB (2014). Indonesia Needs Knowledge Driven Economy to Sustain Growth – Report. https://www.adb.org/news/indonesia-needs-knowledge-driven-economy-sustain-growth-report#:%7E:textItscontributiontotheIndonesian,7.74ofitstotalGDP.%26textKnowledgeeconomiesuseinformationand,andapplyknowledgeforgrowth.

[bib2] Anggraeni Rina (2019). Tren Perusahaan Besar Minati Co-working Spaces Akan Terus Berlanjut. https://ekbis.sindonews.com/read/1392340/34/tren-perusahaan-besar-minati-co-working-space-akan-terus-berlanjut-1554224053.

[bib3] Avdikos V., Merkel J. (2020). Supporting open, shared and collaborative workspaces and hubs: recent transformations and policy implications. Urban Res. Pract..

[bib4] Babb C., Curtis C., McLeod S. (2018). The rise of shared work spaces: a disruption to urban planning policy?. Urban Pol. Res..

[bib5] Bandung Planning Agency (2016). Landuse and Spatial Plan.

[bib6] Brown J. (2017). Curating the “third place”? Coworking and the mediation of creativity. Geoforum.

[bib7] Buksh B., Mouat C.M. (2015). Activating smart work hubs for urban revitalisation: evidence and implications of digital urbanism for planning and policy from South-East Queensland. Aust. Plan..

[bib8] Cabral V., van Winden W. (2016). Coworking: an analysis of coworking strategies for interaction and innovation. Int. J. Knowl.-Based Dev..

[bib9] Capdevila I. (2015). Co-working spaces and the localised dynamics of innovation in Barcelona. Int. J. Innovat. Manag..

[bib10] Carlaw K., Oxley L., Walker P., Thorns D., Nuth M. (2006). Beyond the hype: intellectual property and the knowledge society/knowledge economy. J. Econ. Surv..

[bib11] Davies A.R., Donald B., Gray M., Knox-Hayes J. (2017). Sharing economies: moving beyond binaries in a digital age. Camb. J. Reg. Econ. Soc..

[bib12] Delitheou V., Bakogiannis E., Kyriakidis C. (2019). Urban planning: integrating smart applications to promote community engagement. Heliyon.

[bib13] Di Risio Alberto (2019). The History of Coworking. https://www.coworkingresources.org/blog/history-of-coworking.

[bib14] Dianovita, Khoirunurrofik (2020). The distribution pattern of Co-working space in Jakarta and determinant factors of consumers’ preferences on location decision. IOP Conf. Ser. Earth Environ. Sci..

[bib15] ESRI (2020). How Kernel Density Works. https://pro.arcgis.com/en/pro-app/tool-reference/spatial-analyst/how-kernel-density-works.htm.

[bib16] Florida Richard. (2012). The Rise of the Creative Class.

[bib17] Fost Dan (2008). They’re Working on Their Own, Just Side by Side. https://www.nytimes.com/2008/02/20/business/businessspecial2/20cowork.html.

[bib18] Friedmann J., Miller J. (1965). The urban field. J. Am. Inst. Plan..

[bib19] Garrett L.E., Spreitzer G.M., Bacevice P.A. (2017). Co-constructing a sense of community at work: the emergence of community in coworking spaces. Org. Stud..

[bib20] Geospatial Informasi Bureau (2020). Geospasial Untuk Negeri. https://tanahair.indonesia.go.id/portal-web.

[bib64] Glaeser E.L., Kolko J., Albert S. (2001). Consumer city. J. Econ. Geo..

[bib21] Gore T., Davies H.J., Thöni E., Watts H.D., Sim D. (1989). Review: remaking planning: the politics of urban change in the thatcher years, the reform of local government finance in Britain, local government finance: international perspectives, government policy and industrial change, land use planning and the mediation of urban change: the British planning system in practice. Environ. Plann. C Govern. Pol..

[bib22] Healey J.F. (2012). Statistics: A Tool for Social Research.

[bib23] Hendarman A.F., Tjakraatmadja J.H. (2012). Relationship among soft skills, hard skills, and innovativeness of knowledge workers in the knowledge economy era. Proc. Soc. Behav. Sci..

[bib24] Hicks M., Faulk D.G. (2018). Do entrepreneur-focused facility incentives create economic impacts? Evidence from Indiana. J. Entrepreneurship Publ. Pol..

[bib25] Hsu G.J.Y., Lin Y.-H., Wei Z.-Y. (2008). Competition policy for technological innovation in an era of knowledge-based economy. Knowl. Base Syst..

[bib26] Huliana W., Ellisa E. (2019). Coworking space and cluster spatial relations in the context of Jakarta city spatial structure. IOP Conf. Ser. Earth Environ. Sci..

[bib27] Jamal A.C. (2018). Coworking spaces in mid-sized cities: a partner in downtown economic development. Environ. Plann.: Economy and Space.

[bib28] Kloosterman R.C., Musterd S. (2001). The Polycentric Urban Region: towards a Research Agenda.

[bib29] Kresna Nawa (2016). Melacak Muasal Coworking Spaces di Indonesia. https://tirto.id/melacak-muasal-coworking-space-di-indonesia-b5UK.

[bib65] Kwiatkowski A., Buczynski B. (2011). Coworking: How Freelancers Escape the Coffee Shop Office and Tales of Community from Independents Around the World.

[bib30] Lang LaSalle Jones (2016). A new era of Coworking. https://www.kellerstreetcowork.com/wp-content/uploads/2019/07/JLL-A-New-Era-Of-Coworking-2016.pdf.

[bib31] Law No. 26 of 2007 on Spatial Planning (2007). Laws of the Republic of Indonesia Number 26 of 2007 Concerning Spatial Planning.

[bib32] Mariotti I., Pacchi C., Di Vita S. (2017). Co-working spaces in milan: location patterns and urban effects. J. Urban Technol..

[bib33] Martin C.J. (2016). The sharing economy: a pathway to sustainability or a nightmarish form of neoliberal capitalism?. Ecol. Econ..

[bib34] McCann Philip (2013). Modern Urban and Regional Economics.

[bib35] Mengi O., Bilandzic A., Foth M., Guaralda M. (2020). Mapping Brisbane’s Casual Creative Corridor: land use and policy implications of a new genre in urban creative ecosystems. Land Use Pol..

[bib36] Merkel Janet (2015). Coworking in the city. Ephemera.

[bib37] Moriset B. (2013). Building New Places of the Creative Economy. The Rise of Coworking Spaces.

[bib38] Nakano D., Shiach M., Koria M., Vasques R., Santos E. G. dos, Virani T. (2020). Coworking spaces in urban settings: prospective roles?. Geoforum.

[bib39] Orel M., Dvouletý O., Ratten V. (2020). Transformative changes and developments of the coworking model: a narrative review. Technological Progress, Inequality and Entrepreneurship.

[bib40] Parrino L. (2015). Coworking: assessing the role of proximity in knowledge exchange. Knowl. Manag. Res. Pract..

[bib41] Ranawati N.K. (2020). [Lipkhas] Cerita Patung Bugil Citarum, Tanda Terhubungnya Bandung-Den Haag. https://ayobandung.com/read/2020/01/08/75769/cerita-patung-bugil-citarum-tanda-terhubungnya-bandung-den-haag.

[bib42] Reichenberger I. (2018). Digital nomads – a quest for holistic freedom in work and leisure. Ann. Leisure Res..

[bib43] Richardson L. (2017). Sharing as a postwork style: digital work and the co-working office. Camb. J. Reg. Econ. Soc..

[bib44] Rutten Roel (2003). Knowledge and Innovation in Regional Industry.

[bib45] Rydin Y. (2011). The Purpose of Planning: Creating Sustainable Towns and Cities.

[bib46] Schmidt S., Brinks V. (2017). Open creative labs: spatial settings at the intersection of communities and organizations. Creativ. Innovat. Manag..

[bib47] Schmidt S., Brinks V., Brinkhoff S. (2014). Innovation and creativity labs in Berlin. Z. für Wirtschaftsgeogr..

[bib48] Schürmann Mathias (2013). Coworking Space: Geschäftsmodell für Entrepreneure und Wissensarbeiter.

[bib49] Seto K.C., Pandey B. (2019). Urban land use: central to building a sustainable future. One Earth.

[bib50] Spinuzzi C. (2012). Working alone together: coworking as emergent collaborative activity. J. Bus. Tech. Commun..

[bib51] Stevens Philip, Khan Amir Ullah (2018). Indonesia Needs to Boost its Knowledge Economy to Grow. https://www.thejakartapost.com/academia/2018/10/17/indonesia-needs-to-boost-its-knowledge-economy-to-grow.html.

[bib52] Sutriadi Ridwan, Miftah Ahmad Zaini (2020). Upaya mendorong Kolaborasi menuju pengembangan struktur Ruang Bernuansa knowledge based di Era disrupsi. TATALOKA.

[bib53] Švarc J., Dabić M. (2017). Evolution of the knowledge economy: a historical perspective with an application to the case of europe. J. Knowledge Econ..

[bib54] The Malabar Project (2012). A Monument for J.C. de Groot. https://radiomalabar.wordpress.com/tag/j-c-de-groot/.

[bib55] Thompson B.Y. (2019). The digital nomad lifestyle: (remote) work/leisure Balance, privilege, and constructed community. Int. J. Sociol. Leisure.

[bib56] Tobler W.R. (1970). A Computer Movie Simulating Urban Growth in the Detroit Region.

[bib57] UNESCO (2015). Bandung. https://en.unesco.org/creative-cities/bandung.

[bib58] Wagner W.E. (2015). Using IBM SPSS Statistics for Research: Methods and Social Science Statistics.

[bib59] Waters-Lynch J.M., Potts J., Butcher T., Dodson J., Hurley J. (2016). Coworking: a transdisciplinary overview. SSRN Electron. J..

[bib60] Weijs-Perrée M., van de Koevering J., Appel-Meulenbroek R., Arentze T. (2019). Analysing user preferences for co-working space characteristics. Build. Res. Inf..

[bib61] Wicklin R. (2016). How to Visualize a Kernel Density Estimate. https://blogs.sas.com/content/iml/2016/07/27/visualize-kernel-density-estimate.html.

[bib62] Yigitcanlar T. (2010). Making space and place for the knowledge economy: knowledge-based development of Australian cities. Eur. Plann. Stud..

[bib63] Zhou Yaoyi (2019). Coworking as an Emerging Urban Lifestyle: Location Analysis of Coworking Spaces in Manhattan. https://cuny.manifoldapp.org/read/untitled-cb0c686f-43b1-401c-8211-7c8c79735fcf/section/05e9f179-5e4e-4d49-80ed-00ba9067f972.

